# Dosimetric comparison of helical tomotherapy, VMAT, fixed-field IMRT and 3D-conformal radiotherapy for stage I-II nasal natural killer T-cell lymphoma

**DOI:** 10.1186/s13014-017-0812-1

**Published:** 2017-04-27

**Authors:** Xianfeng Liu, Erliang Huang, Ying Wang, Yanan He, Huanli Luo, Mingsong Zhong, Da Qiu, Chao Li, Han Yang, Guanglei He, Juan Zhou, Fu Jin

**Affiliations:** 1 Department of Radiation Oncology, Chongqing Cancer Institute & Hospital & Cancer Center, Shapingba District, Chongqing, 400030 China; 2 Department of Medical Equipment Guangzhou Women and Children’s Medical Center, Guangzhou, 510623 People’s Republic of China; 3 Forensic Identification Center, College of Criminal Investigation, Southwest University of Political Science and Law, Chongqing, People’s Republic of China

**Keywords:** Helical tomotherapy, VMAT, IMRT, Nasal natural killer/T-cell lymphoma, Dosimetry

## Abstract

**Background:**

The aim of this study was to compare radiotherapy plans for Stage I-II nasal natural killer/T-cell lymphoma (NNKTL) using helical tomotherapy (HT), volumetric-modulated arc therapy (VMAT), Fixed-Field intensity-modulated radiotherapy (IMRT), and three-dimensional conformal radiotherapy (3D-CRT).

**Methods:**

Eight patents with Stage I-II NNKTL treated with IMRT were re-planned for HT, VMAT (two full arcs), and 3D-CRT. The quality of target coverage, the exposure of normal tissue and the efficiency of radiation delivery were analyzed.

**Results:**

HT showed significant improvement over IMRT in terms of D_98%_, cold spot volume and homogeneity index (HI) of planning target volume (PTV). VMAT provided best dose uniformity (*p* = 0.000) to PTV, while HT had best dose homogeneity among the four radiotherapy techniques (*p* = 0.000) to PTV. VMAT obviously reduced treatment time (*p* = 0.010; 0.000) compared to HT and IMRT. Mean dose of left and right optic nerve was significantly reduced by IMRT compared to HT (19.86%, *p* = 0.000; 21.40%, *p* = 0.002) and VMAT (8.97%, *p* = 0.002; 9.35%, *p* = 0.001), and maximum dose of left lens of VMAT increased over the HT (36.25%, *p* = 0.043) and IMRT (40.65%, *p* = 0.001).

**Conclusion:**

The unexpected results show that both HT and VMAT can achieve higher conformal treatment plans while getting worse organs at risk (OARs) sparing than IMRT for patients with Stage I-II NNKTL. VMAT requires the shortest delivery time, and IMRT delivers the lowest dose to most OARs. The results could provide guidance for selecting proper radiation technologies for different cases.

## Background

The incidence of NNKTL accounts for 2–10% of the cases of primary non-Hodgkin’s disease [1]. It occurs more commonly in Asians, Mexicans and South Americans than in Western populations [2–5]. About 80% of cases diagnosed with localized disease are of stage I-II [6], but treatment outcomes in the cases remain unsatisfying. Recent studies have showed that primary radiotherapy is superior to chemotherapy alone or primary chemotherapy [4, 7, 8]. However, the local recurrence rate remains as high as 41–50%, and the overall survival rate is lower than 50% for patients with stage I-II NNKTL after radiotherapy [9–11]. Retrospective studies have reported that improper radiation fields and deficiency of radiation dose for target volume are two of the main causes accounting for treatment failure [10–12]. Radiotherapy is highly recommended as the primary therapy for stage I-II NNKTL, so the establishment of feasible and optimal patterns of radiotherapy is critical. Therefore, the optimal radiotherapeutic techniques, including radiation dose, target volume, and treatment plans, still deserve further study.

Though highly conformal radiation techniques, such as HT, IMRT, and VMAT have been widely studied in head-and-neck cancer, there are only several reported investigations to NNKTL with these techniques. Shen et al. [13] studied 94 patients with stage I-II NNKTL and confirmed that IMRT had shown dosimetric advances in target dose sculpturing and OARs sparing, and yet particularly resulted in complex technical problems and extended treatment time. In recent years, radiotherapy techniques have dramatically improved. Helical tomotherapy delivers IMRT treatment with 64 pneumatically driven leaves of multi-leaf collimator (MLC), selectable fixed jaws and 360° gantry rotation while the patient couch is translating. VMAT, a variable-speed rotational treatment paradigm, delivers IMRT treatment with less monitor units (MUs), less treatment time, varying dose rates and dynamic MLC. Recently, comparisons have been published on radiotherapy techniques in pairs for treating patients with stage I-II NNKTL, indicating some potential dosimetric disadvantages and advantages [13–15]. Yet, this study aims to further assess treatment plans utilizing 4 kinds of radiotherapy techniques: HT, VMAT, IMRT and 3D-CRT for treatment of stage I-II NNKTL.

## Methods

### Patients and materials

Eight consecutive patients with localized Stage I-II NNKTL were treated with Fixed-Field IMRT at our Institution between September 2010 and March 2013, seven patients with Stage I and one patient with Stage II. All patients were free of distant metastases and had not received prior radiotherapy.

Philips Brillicance Big Bore computed tomography (CT) (Philips, Holland) simulation was used to scan at 3 mm slice thickness with a scan scope from the vertex of the skull to the inferior margin of the clavicular heads on supine position. The CT images were imported to the Eclipse treatment planning system (Varian Medical Systems, Version 11.0, Inc.) and prepared for contouring.

**Fig. 1:**
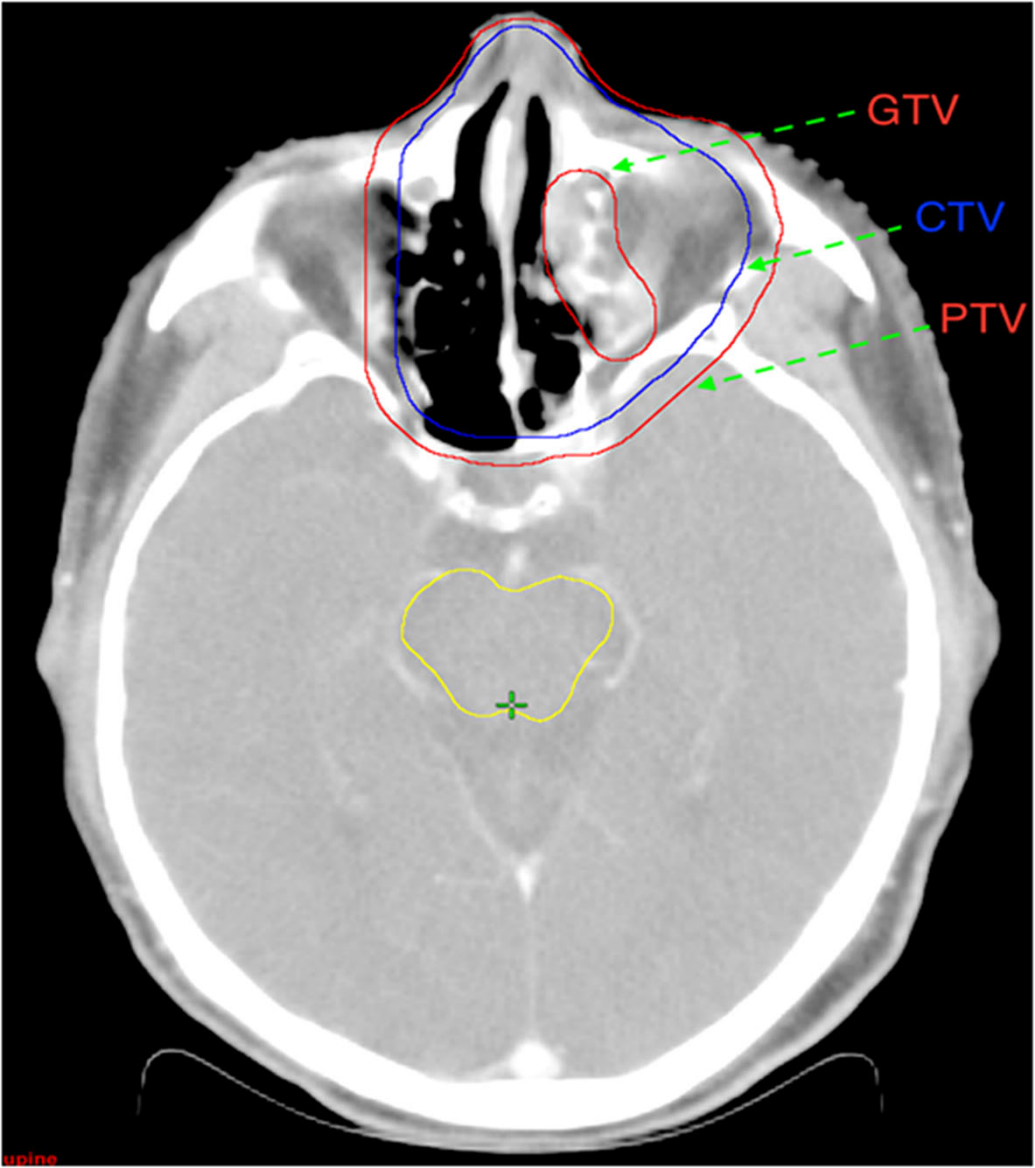
The definition of the target volume

According to the report by the International Commission on Radiation Units and Measurement, the gross tumor volume (GTV) contains the primary tumor and regional metastatic lymph nodes, which are identified by CT or magnetic resonance imaging (MRI), physical examination, and endoscopic examinations. For limited IE phase, if the tumor is confined to one side of the nasal cavity, without invasion of adjacent tissues or organs, the clinical target volume (CTV) should include ipsilateral nasal cavity, ipsilateral anterior ethmoid sinus and maxillary sinus. If the tumor has invaded nasal cavity or nasal septum, the CTV should include bilateral nasal cavity, bilateral anterior ethmoid sinus and bilateral maxillary sinus. For extensive IE period, the CTV should be extent to the adjacent tissues or organs. For phase IIE, the CTV also includes bilateral neck lymph drainage area, in addition to irradiating the above tissue structure. The planning target volume (PTV) is obtained from the respective CTV adding 3 mm margin with all expansion to offset setup uncertainties. The mean volume of PTV was 286.4 cm^3^, ranging from 60.7 cm^3^ to 471.3 cm^3^. Figure 1 shows an example of the target volume contouring for a patient.

### Radiotherapy plans

For each case, four different planning techniques were adopted: HT, VMAT, Fixed-Field IMRT, and 3D-CRT. The HT plans were designed on tomotherapy treatment planning system with 6 MV photon beams [15] and optimized via least squares optimization method [16]. VMAT, Fixed-Field IMRT, and 3D-CRT plans were designed on the Varian Eclipse treatment planning system with 6 MV photon beams generated by Varian IX linear accelerator. For all Eclipse plans, Dose–Volume Optimizer (DVO) and Progressive Resolution Optimizer (PRO) algorithms were used for IMRT and VMAT optimizations, respectively [17]. Anisotropic analytical algorithm(AAA) was applied for final dose calculations [18].

All plans were optimized with an addition of a 0.5 cm bolus. The right and left bounds of the bolus lie on the inner edges of the paropias on both sides, while the lower and upper bounds lie on the lower edge of the palate, and the upper edge of the frontal sinus respectively.

- *HT*
  A field width of 2.5 cm, pitch values of 0.287, modulation factor of 3 and fine dose calculation grid was used.
- *VMAT*
  The VMAT plan included two coplanar arcs of 360°, with the collimator rotation of 45° and 315° respectively, and the couch rotation set to 0°. The plans were optimized with a maximum dose rate (DR) of 600 MUs/min.
- *Fixed-field IMRT*
  Fixed-field IMRT plans contained 9 equally distributed coplanar fields, with the following gantry angles: 200°/240°/280°/320°/0°/40°/80°/120°/160°. Given the lenses and optic nerves close to the PTV, the angle of collimator and the position of jaw in some field should be adjusted. Specifically, for the treatment fields with gantry angle 0°, two fields, lower and upper fields, are obtained at the interface 0.5 cm below the lower edge of the lens and the angle of collimator is set to 0°. A fixed DR of 300 MUs/min and dynamic sliding-window IMRT delivery technique were used to optimize the delivered dose.
- *3D-CRT*
  3D-CRT plans contained three half fields, one anterior and two bilateral fields, are obtained at the interface 0.5 cm below the lower edge of the lens. The anterior fields radiated the upper part of PTV, and the two bilateral fields radiated the lower part of PTV. The collimator of all fields is set to 0°, the gantry angle of the anterior, and two bilateral fields are set to 0°, 270° and 90° respectively. To solve the dose uniformity, a 30° physical wedge filter was added to the two bilateral fields.

The prescribed dose of the PTV was 50 Gy in total, 25 fractions over 5 weeks. For all treatment plans, the prescribed 95% isodose covered at least 95% of the PTV, and the percentage volume of PTV receiving greater than 107% of the prescription was limited to 2% [14, 19].

Contoured OARs were lenses, eyes, optic chiasm, optic nerves, brainstem, spinal cord and parotid glands. All CT images and contoured structures in the Eclipse treatment planning system were transmitted to the tomotherapy treatment planning system (Tomotherapy, Madison, WI). Dose constraints for the four types of plans were adopted and slightly modified from the Radiation Therapy Oncology Group (RTOG) 0615 Protocol [20]. The dose constraint for the brainstem and lens was reduced to 50 Gy and 15 Gy, respectively.

Planning objective for other OARs was defined as follows: maximum dose (D_max_) of optic chiasm, optic nerves, eyes were limited to < 50 Gy; D_max_ of spinal cord was limited to 45 Gy; mean dose (D_mean_) of parotid glands was limited to < 26 Gy.

### Treatment plan evaluation

**Fig. 2:**
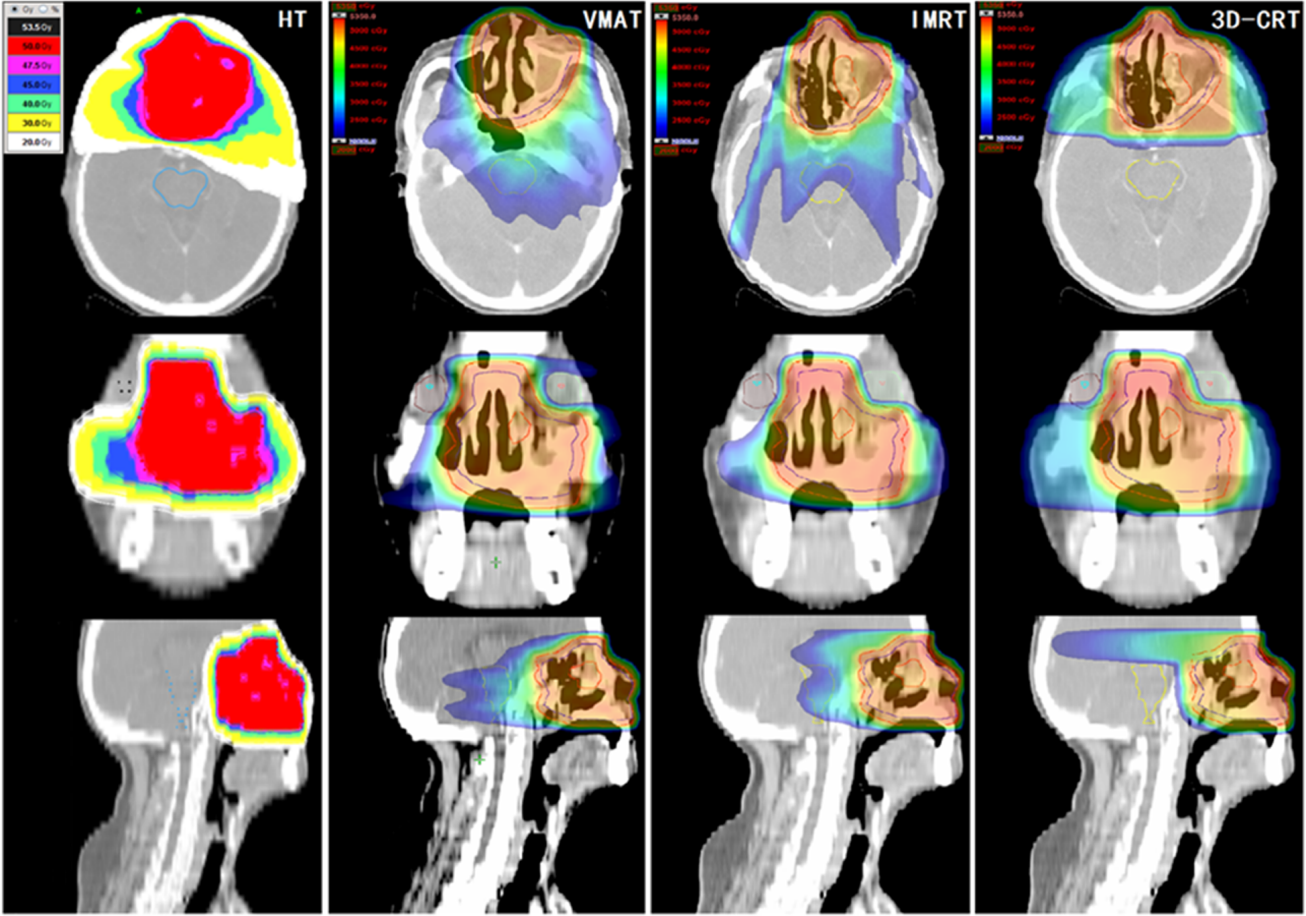
Example of the dose distribution using HT, VMAT, IMRT and 3D-CRT for the same patient

**Fig. 3:**
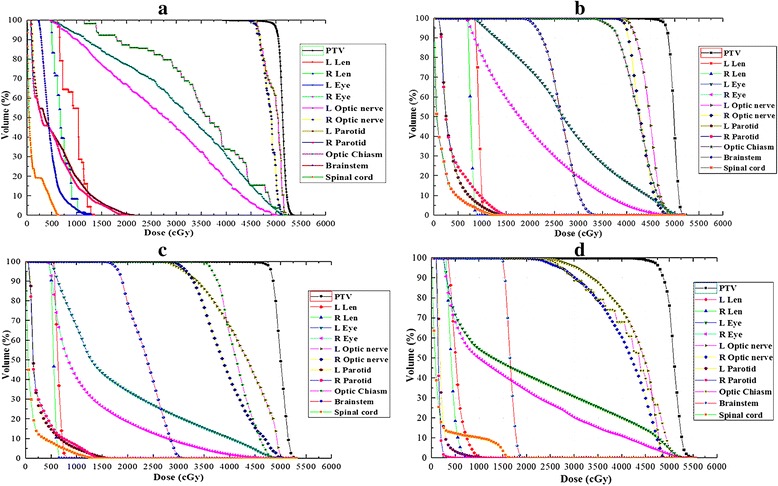
DVH for (**a**) HT, (**b**) VMAT, (**c**) IMRT and (**d**) 3D-CRTplans for the same patient

The data from the Dose-Volume Histogram (DVH) obtained from all the plans were analyzed. Representative dose distribution and DVHs for four types of techniques are shown in Figs. 2 and 3. The plan comparisons were focusing on the following items.

PTV Coverage: D_98%_ and D_2%_ (dose received by 98%, and 2% of the PTV volume respectively) were defined as the near-minimum and near-maximum dose of PTV. The D_mean_ to the PTV and the percentage of PTV covered by ≥ 95% of the prescribed dose (V_95%_) were also used. Cold spot volume (the percentage volumes of PTV receiving less than 93% of the prescribed dose) should be less than 1%. Conformal index (CI) of PTV was derived from the prescription isodose volume divided by the PTV volume. A value of CI approaching 1 indicates the conformity of PTV is fine. Homogeneity index (HI) of PTV calculated with D_5%_ minus D_95%_, divided by D_mean_, where D_x%_ is the minimum dose delivered to x% of the PTV, D_mean_ is the mean dose of the PTV. Higher HI represented poorer homogeneous irradiation of the PTV.

Organs at Risk: for each patient, analysis was performed for the D_max_ and D_mean_ of OARs, including left and right eye, left and right lens, left and right optic nerve, optic chiasm, spinal cord, brainstem, left and right parotid gland.

Monitor Units and Treatment Time: the total MU and treatment time were used to compare the four kinds of technology. Treatment time was calculated from beam-on to the end of total MU delivery, inclusive of the time for gantry rotation and radiation delivery. Because the time of install and uninstall physical wedge filter could not be precisely measured, the comparison of treatment time did not contain 3D-CRT.

### Statistical analysis

Paired *t*-test was used to determine if there was a significant difference in each of the parameters examined with the SPSS statistical software (SPSS, Chicago, IL, USA). A *p* < 0.05 was considered statistically significant.

## Results

### PTV coverage

The data analysis of all plans for PTV was performed with the DVH, the PTV coverage parameters:D_98%_, D_2%_, D_mean_, cold spot volume, CI_95%_ and HI were compared. HT could provide better D_98%_ and HI than VMAT, IMRT and 3D-CRT (*p* < 0.05), while achieving worse D_2%_ and CI_95%_ than VMAT and IMRT (*p* < 0.05) but better than 3D-CRT (*p* < 0.05). Compared with IMRT, cold spot volume (%) of PTV with HT and VMAT decreased by 52.27% (*p* = 0.036) and 30.68% (*p* = 0.047). The CI_95%_ was better with VMAT (1.075 ± 0.008) than with HT (1.234 ± 0.024) and 3D-CRT (1.493 ± 0.046) (*p* < 0.05). The findings from DVH analysis on PTV are listed in Table 1.

**Table 1:** Results of dosimetric comparison for PTV from DVH ($\overline{x}\pm S$)

Parameters	HT	VMAT	IMRT	3D-CRT	*p*-Value					
					HT VS. VMAT	HT VS. IMRT	HT VS. 3D-CRT	VMAT VS. IMRT	VMAT VS. 3D-CRT	IMRT VS. 3D-CRT
D_98%_ (Gy)	49.12 ± 0.19	47.43 ± 0.18	47.20 ± 0.10	47.50 ± 0.24	0.000	0.000	0.002	0.181	0.766	0.275
D_2%_ (Gy)	52.42 ± 0.17	51.31 ± 0.11	51.62 ± 0.19	54.80 ± 0.47	0.000	0.004	0.001	0.055	0.000	0.000
D_mean_ (Gy)	51.41 ± 0.15	49.75 ± 0.10	49.72 ± 0.18	50.98 ± 0.12	0.000	0.000	0.056	0.862	0.000	0.001
V_95%_(%)	99.35 ± 0.19	97.82 ± 0.44	97.10 ± 0.25	97.74 ± 0.48	0.015	0.000	0.024	0.150	0.863	0.279
Cold spot volume (%)	0.42 ± 0.45	0.61 ± 0.37	0.88 ± 0.33	0.95 ± 0.22	0.367	0.036	0.107	0.047	0.105	0.773
CI_95%_	1.234 ± 0.024	1.075 ± 0.008	1.090 ± 0.007	1.493 ± 0.046	0.000	0.000	0.000	0.140	0.000	0.000
HI	0.043 ± 0.002	0.058 ± 0.003	0.065 ± 0.004	0.103 ± 0.008	0.000	0.000	0.000	0.066	0.000	0.002

### OARs

The average dose to the OARs are listed in Table 2. In comparison with HT, VMAT and IMRT reduced the maximum dose of optic chiasm (*p* = 0.016; *p* = 0.020), left optic nerve (0.007; 0.033), right optic nerve (0.002; 0.018) and left parotid (0.031, 0.010). Similarly, compared with VMAT, the maximum dose to left lens with HT and IMRT was significantly decreased by 26.61 and 28.90% (*p* = 0.043; 0.001), the maximum dose to right lens with IMRT was greatly reduced by 24.97% (*p* = 0.000), and the maximum dose of brainstem of HT and IMRT was decreased by 20.44 and 7.54% (*p* = 0.044; 0.000).

**Table 2:** Results of dosimetric comparisons for OARs from DVH ($\overline{x}\pm S$)

Parameters		HT	VMAT	IMRT	3D-CRT	*p*-Value					
						HT VS. VMAT	HT VS. IMRT	HT VS. 3D-CRT	VMAT VS. IMRT	VMAT VS. 3D-CRT	IMRT VS. 3D-CRT
Optic chiasm	D_max_ (Gy)	48.48 ± 1.52	47.13 ± 1.33	46.50 ± 1.17	48.13 ± 0.94	0.016	0.020	0.761	0.174	0.340	0.162
	D_mean_ (Gy)	38.01 ± 2.95	37.75 ± 2.44	37.77 ± 1.57	40.75 ± 1.60	0.880	0.908	0.210	0.984	0.108	0.046
Left optic nerve	D_max_ (Gy)	49.43 ± 2.42	46.62 ± 3.12	47.01 ± 3.31	46.87 ± 3.37	0.007	0.033	0.053	0.264	0.724	0.834
	D_mean_ (Gy)	45.56 ± 2.86	41.81 ± 3.59	38.01 ± 3.66	36.92 ± 4.98	0.002	0.000	0.012	0.007	0.049	0.609
Right optic nerve	D_max_ (Gy)	50.32 ± 0.75	48.02 ± 1.02	48.23 ± 1.09	49.08 ± 1.22	0.002	0.018	0.281	0.774	0.300	0.272
	D_mean_ (Gy)	45.95 ± 1.83	42.02 ± 2.23	37.85 ± 2.73	36.70 ± 4.39	0.001	0.002	0.024	0.058	0.152	0.570
Left eye	D_max_ (Gy)	45.74 ± 2.81	45.39 ± 4.24	44.15 ± 3.79	51.35 ± 3.12	0.859	0.329	0.003	0.264	0.005	0.000
	D_mean_ (Gy)	21.36 ± 2.23	22.43 ± 2.64	15.15 ± 2.17	22.01 ± 5.09	0.550	0.008	0.881	0.001	0.914	0.072
Right eye	D_max_ (Gy)	44.90 ± 2.17	43.56 ± 2.31	39.78 ± 4.19	49.71 ± 3.45	0.213	0.090	0.084	0.116	0.007	0.000
	D_mean_ (Gy)	20.49 ± 2.12	19.94 ± 2.37	13.34 ± 2.34	19.45 ± 5.10	0.745	0.005	0.805	0.000	0.881	0.070
Left lens	D_max_ (Gy)	8.00 ± 1.09	10.90 ± 1.15	7.75 ± 0.70	15.27 ± 6.30	0.043	0.815	0.303	0.001	0.511	0.254
	D_mean_ (Gy)	5.41 ± 0.67	8.93 ± 0.72	6.39 ± 0.52	11.40 ± 6.65	0.002	0.228	0.404	0.000	0.719	0.460
Right lens	D_max_ (Gy)	7.43 ± 0.89	9.33 ± 0.44	7.00 ± 0.49	13.92 ± 6.53	0.118	0.693	0.373	0.000	0.488	0.301
	D_mean_ (Gy)	4.83 ± 0.41	8.05 ± 0.46	5.92 ± 0.46	10.98 ± 6.54	0.000	0.083	0.382	0.000	0.662	0.450
Spinal cord	D_max_ (Gy)	18.94 ± 4.76	21.67 ± 5.46	19.02 ± 4.30	19.65 ± 5.36	0.236	0.968	0.806	0.086	0.615	0.852
	D_mean_ (Gy)	2.70 ± 0.76	2.43 ± 0.67	2.20 ± 0.63	2.23 ± 0.51	0.363	0.060	0.191	0.024	0.438	0.895
Brainstem	D_max_ (Gy)	30.60 ± 4.41	38.46 ± 1.70	35.56 ± 1.73	33.74 ± 4.21	0.044	0.168	0.090	0.000	0.189	0.601
	D_mean_ (Gy)	19.15 ± 3.27	25.98 ± 1.48	25.24 ± 1.62	20.72 ± 3.05	0.097	0.081	0.652	0.471	0.191	0.181
Left parotid	D_max_ (Gy)	29.48 ± 4.46	26.34 ± 5.31	25.78 ± 4.60	34.16 ± 6.78	0.031	0.010	0.212	0.622	0.015	0.039
	D_mean_ (Gy)	14.51 ± 3.02	11.51 ± 2.97	10.87 ± 2.72	13.66 ± 4.05	0.007	0.000	0.577	0.230	0.256	0.133
Right parotid	D_max_ (Gy)	25.27 ± 4.77	22.38 ± 5.59	23.68 ± 5.51	28.48 ± 7.38	0.031	0.232	0.454	0.095	0.110	0.156
	D_mean_ (Gy)	12.20 ± 4.06	11.08 ± 3.53	10.31 ± 3.14	13.14 ± 4.63	0.372	0.280	0.578	0.243	0.220	0.144

### MUs and treatment time

The MUs and treatment time of the four treatment techniques are shown in Table 3. The mean monitor units of HT, VMAT, IMRT and 3D-CRT were 3345.63, 639.88, 1257.13 and 610.50. Compared with IMRT, the mean MUs of VMAT and 3D-CRT were significantly reduced by 49.10 and 51.44% (*p* = 0.000; 0.000). The mean treatment time of HT, VMAT and IMRT were 238.98, 191.85 and 436.13 s. In comparison with IMRT, the mean treatment time of HT and VMAT were greatly decreased by 45.20 and 56.01% (*p* = 0.000; 0.000).

**Table 3:** Results of MUs and treatment time ($\overline{x}\pm S$)

Parameters	HT	VMAT	IMRT	3D-CRT	*p*-Value					
					HT VS. VMAT	HT VS. IMRT	HT VS. 3D-CRT	VMAT VS. IMRT	VMAT VS. 3D-CRT	IMRT VS .3D-CRT
MUs	3345.63 ± 207.02	639.88 ± 28.92	1257.13 ± 59.61	610.50 ± 8.09	0.000	0.000	0.000	0.000	0.377	0.000
Time (s)	238.98 ± 14.15	191.85 ± 1.24	436.13 ± 11.34	/	0.010	0.000	/	0.000	/	/

## Discussion

The report about dosimetric comparison of various radiation technologies for stage I-II NNKTL is rare, so present study reporting a comparison among HT, VMAT, IMRT and 3D-CRT plans for treating stage I-II NNKTL is significant. The goal of this study is overall estimate of dosimetric properties in four types of treatment plans, and provides the direction for technologies selecting in auto-planning design of stage I-II NNKTL.

Previous studies [13, 15] confirmed that IMRT and HT have demonstrated significantly steeper dose gradient around the target than 3D-CRT, allowing high quality of conformal avoidance and dose sculpturing for improving the gain ratio of radiotherapy and the local control of the tumor. Comparing with other studies, we generally found that IMRT and HT offered similar results in PTV coverage and dose conformity, and all plans met the clinical demand, but 3D-CRT was distinctly worse in tumor coverage and dosimetric accuracy. The main disadvantage of IMRT was that it spent relatively long time on radiation delivery, increasing patients’ discomfort and the probability of patents’ moving during treatment. HT had an obvious characteristic of sharp dose gradients, with the highest D_98%_, the largest V_95%_ and the optimal HI.

Compared with IMRT, VMAT was one of the hottest issues in recently years. VMAT therapy in head and neck cancers has demonstrated excellent target coverage with highly conformal dose, improvements in OARs sparing and delivery time [21–23]. However, only our study utilized VMAT for stage I-II NNKTL [14] up to present. In the study, we observed VMAT (two coplanar full arcs) over 9-field IMRT for Stage I-II NNKTL. We found that the cold spot volume, homogeneity and conformity with VMAT plan were slightly superior to that with the IMRT plan (cold spot volume = 0.56 ± 0.11 of VMAT versus 0.93 ± 0.013 of IMRT; HI = 0.060 ± 0.003 of VMAT versus 0.069 ± 0.004 of IMRT; CI = 1.069 ± 0.007 of VMAT versus 1.087 ± 0.006 of IMRT). In the current study, we found that HT and IMRT achieved better D_2%_, CI_95%_ and HI than 3D-CRT did. HT and VMAT provided better D_98%_, cold spot volume and HI than IMRT. VMAT provided the optimal CI_95%_ and radiation delivery time, which was confirmed by much evidence in previous studies [24–28]. With this benefit, VMAT can improve the clinical throughput and allows more time to perform systematic image guidance. HT achieved the optimal D_98%_, and HI among the four technologies, while D_2%_ and CI_95%_ with HT was slightly inferior to that with VMAT and IMRT, but superior to that with 3D-CRT. However, it should be mentioned that if 100% isodose surface volume is selected as reference volume to define conformal index, maybe HT could obtain the optimal CI.

Nevertheless, we noticed that IMRT achieved the optimal sparing of nearby critical tissues in our study where the dose to OARs did not exceed the limits among the four treatment technologies. HT and 3D-CRT had higher maximum and mean radiation dose in most parts of the OARs, and VMAT had higher D_max_ of the brainstem and D_mean_ of the left eye. Ke Sheng et al. [29] studied 10 oropharyngeal carcinoma patients and proved HT superior to IMRT in normal tissue sparing; especially reduced the risk for complication of parotid glands. Similar results were also reported by Anders Bertelsen et al. [26] who studied 25 patients with oropharyngeal or hypopharyngeal carcinoma and demonstrated that VMAT offered equivalent or improved sparing of OARs compared with IMRT. In our study, HT and VMAT obtained better quality of dose distributions than IMRT did, though more prejudicing the OARs. This implies that pursuit of a best quality plan to the utmost possibly had no advantage. There are some findings about OARs in our study disagreement with what was reported by Ke Sheng et al. [29] and Anders Bertelsen et al. [26]. This is because that oropharyngeal and hypopharyngeal differ from NNKTL in the location of OARs and PTV. In general, the primary site of NNKTL is nasal tumor or invasion in nasal cavity, and the target volume must contain the maxillary sinus, nasal cavity and ethmoidal sinus, which means that the target volume is much closer to lenses, optic chiasm, optic nerves and eyes. As HT and VMAT pursue improved CI or HI in the target volume, the dose to OARs will inevitably increase. Therefore, compared with IMRT, HT and VMAT obtained not only a slightly better CI or HI in the target volume, but also higher dose to OARs.

Plan design for NNKTL is one of the most complex tasks due to the complexity of the tumor shape and surrounding OARs, resulting in a time consuming design process. Recently, two innovative approaches have been implemented to get automatically of the optimal plan: knowledge -based model (RapidPlan from Eclipse [30]) and template-based model (Erasmus-iCycle works with Monaco [31] and Autoplan from Pinnacle3 [32]). For the knowledge-based planning, based on contoured anatomy, the dose distribution of new patients is estimated, utilizing the dose and patient anatomy information from existing plans. In this study, the results indicate that IMRT specializes in the protection of OARs, HT and VMAT have advantage over getting better target quality, so in the process of knowledge-based auto-planning, IMRT should be adopted to protect the OARs, while both HT and VMAT are used to acquire optimal target quality. In addition, the template-based model optimizes treatment plans automatically, utilizing settings with prioritization and compromise of OARs, which is associated with location and dose constraints of PTV and OARs. In this article, the location correlation between PTV and OARs is not involved in and will be further expanded in future studies.

## Conclusion

For Stage I-II NNKTL, both HT and VMAT showed slight improvements in target quality compared with IMRT, which was much better than 3D-CRT. However, HT could offer the largest V_95%_ and VMAT featured with lower MUs and shorter delivery time but with higher dose to OARs than IMRT.

## Abbreviations

3D-CRT: Three-dimensional conformal radiotherapy
AAA: Anisotropic analytical algorithm
CI: Conformal index
CTV: Clinical target volume
D_max_: Maximum dose
D_mean_: Mean dose
DR: Dose rate
DVH: Dose-volume histogram
DVO: Dose–volume optimizer
GTV: Gross tumor volume
HI: Homogeneity index
HT: Helical tomotherapy
IMRT: Fixed-field intensity-modulated radiotherapy
MLC: Multi-leaf collimator
MUs: Monitor units
NNKTL: Nasal natural killer/T-cell lymphoma
OARs: Organs at risk
PRO: Progressive resolution optimizer
PTV: Planning target volume
RTOG: Radiation therapy oncology group
VMAT: Volumetric-modulated arc therapy
